# Robust Diabatic Grover Search by Landau–Zener–Stückelberg Oscillations

**DOI:** 10.3390/e21100937

**Published:** 2019-09-25

**Authors:** Yosi Atia, Yonathan Oren, Nadav Katz

**Affiliations:** 1 The Rachel and Selim Benin School of Computer Science and Engineering, The Hebrew University, Jerusalem 91904, Israel; or.yonatan@gmail.com; 2 The Racah Institute of Physics, The Hebrew University, Jerusalem 91904, Israel; katzn@phys.huji.ac.il

**Keywords:** adiabatic quantum computing, quantum algorithms, quantum error correction, quantum two-level systems, coherent destruction of tunneling, quantum control

## Abstract

Quantum computation by the adiabatic theorem requires a slowly-varying Hamiltonian with respect to the spectral gap. We show that the Landau–Zener–Stückelberg oscillation phenomenon, which naturally occurs in quantum two-level systems under non-adiabatic periodic drive, can be exploited to find the ground state of an *N*-dimensional Grover Hamiltonian. The total runtime of this method is $O(\sqrt{2^{n}})$, which is equal to the computational time of the Grover algorithm in the quantum circuit model. An additional periodic drive can suppress a large subset of Hamiltonian control errors by using coherent destruction of tunneling, thus outperforming previous algorithms.

## 1. Introduction

Adiabatic Quantum Computation (AQC) [1,2] is a computational model motivated by the adiabatic theorem. The theorem states that, if a system is prepared in the ground state of an initial Hamiltonian, and the Hamiltonian slowly varies over time, then the system will remain close to the instantaneous ground state [3,4]. By encoding a solution for a computational problem in the ground state of the final Hamiltonian, one can exploit this phenomenon to produce the aforementioned ground state, and thus produce a solution to the problem. The maximal rate of change allowed for such evolution usually scales with the square of the energy gap between the ground state and the first excited state [1].

The Grover problem [5], also known as The Unstructured Search Problem, is one of the few problems solvable by a native adiabatic algorithm, which achieves the same performance as the best possible algorithm in the circuit model [6] (see also [7,8,9]). The input to the problem is an *n* qubit Hamiltonian $H_{p}$, which can only be used as a black box, meaning it can be switched on or off:

(1) $$H_{p}=I_{N}-|y\rangle \langle y|,$$

wherein $I_{N}$ is the N×N identity matrix with $N=2^{n}$, and we use dimensionless units (see Appendix A). The problem is to find the unknown string *y*. Grover’s algorithm solves the problem in time $O(\sqrt{N})$, which is a quadratic improvement to any classical algorithm. The problem is comparable to finding the ground state of a known Hamiltonian, whose ground state is computationally hard to find and, therefore, can be considered *computationally unknown* [10].

An adiabatic algorithm for the search problem was first suggested by Farhi et al. [1]. The system is initialized to a symmetric superposition of the computational basis states, denoted |u〉=|+⋯+〉, and then evolves by the time-dependent Hamiltonian:

(2) $$H_{G}\left(s(t)\right)=\left(1-s\left(t\right)\right)\cdot \left(I_{N}-|u\rangle \langle u|\right)+s\left(t\right)\cdot \left(I_{N}-|y\rangle \langle y|\right),$$

wherein the control function $s\left(t\right):\left[t_{i},t_{f}\right]\to \left[0,1\right]$ is initialized to 0 and increases monotonically in time to 1. The minimal gap for *n* qubit systems is $\Delta =\sqrt{2^{-n}}$. Evolving with a linear s(t) requires $O(2^{n})$ time, while a specially tailored control function, whose rate matches the instantaneous spectral gap, generates the ground state of $H_{p}$ in the optimal time, $O(\sqrt{2^{n}})$ [11,12].

In this work, we introduce a *diabatic* algorithm for the Grover problem, denoted algorithm A, whose performance matches the optimal adiabatic and circuit model algorithms [5,6,12], by setting s(t)=(1−Acos(ωt))/2 when ω≫Δ. The system passes the minimal gap multiple times diabatically and is effectively evolving by a Landau–Zener–Stükelberg (LZS) Hamiltonian [13,14,15,16,17] (which is sometimes referred to as the Landau–Zener–Stükelberg–Majorana Hamiltonian [18]). In algorithm B, we add an oscillating term $B\cos\left(\omega t\right)|u\rangle \langle u|$ that yields improved robustness to Hamiltonian control errors relative to previous algorithms [5,12].

## 2. Background

### 2.1. LZS Hamiltonians

We start by analyzing the LZS Hamiltonian for a generic two-level system with the bare states |0〉,|1〉, closely following the treatment of [19]:

(3) $$\begin{array}{c} H_{\mathrm{LZS}}\left(t\right):=\frac{1}{2}\left(-A\cos\left(\omega t\right)\sigma_{z}-\Delta \sigma_{x}\right)=\frac{1}{2}\left[\begin{array}{cc} -A\cos(\omega t) & -\Delta \\ -\Delta & A\cos(\omega t) \end{array}\right]. \end{array}$$

**Figure 1:**
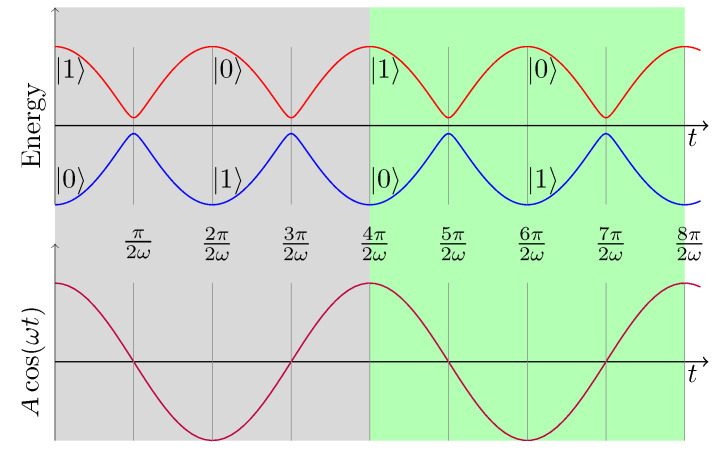
(**top**) The instantaneous eigenvalues of $H_{\mathrm{LZS}}\left(t\right)$; and (**bottom**) the drive Acos(ωt). Avoided crossings occur at $t=\pi (k+\frac{1}{2})/\omega$ for integer *k*, when cos(ωt)=0. Each period of the drive (gray or green background) contains a double crossing. Note that the ground state and the excited state alternate at every avoided crossing.

The sinusoidal drive causes the Hamiltonian to exhibit avoided level crossings at $t=\pi (k+\frac{1}{2})/\omega$ for k∈N with a minimal energy gap of Δ (see Figure 1).

**Figure 2:**
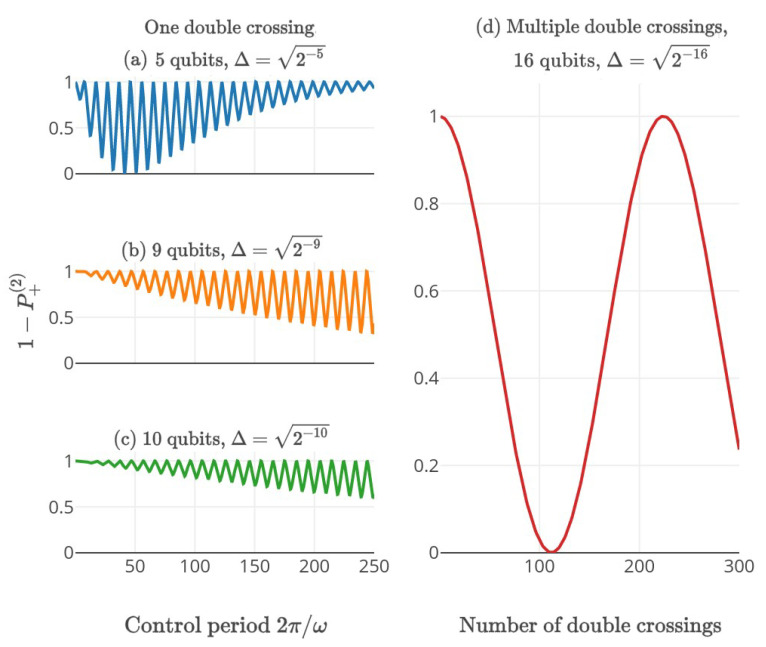
Numerical simulation of LZS oscillations solving the Grover problem where the system is initialized to the ground state at t=0. (**a**–**c**) The ground state population after a double crossing with different ω and gaps. This probability reaches 1 both for ω≫A and for $\omega \ll \Delta^{2}/A$ (only visible in (**a**)). For the first limit, the system is almost unperturbed. However, in second limit, the process is adiabatic and the system follows the instantaneous ground state and returns to its initial state. While the rotating wave approximation holds (ω≫Δ), the ground state population after a double crossing is $P_{+}^{\left(2\right)}=\Delta \left|J_{0}\left(\frac{A}{\omega }\right)\right|\cdot 2\pi /\omega$. The zeros of the Bessel function correspond to coherent destruction of tunneling, wherein $1-P_{+}^{\left(2\right)}=1$ in the graph. The approximation fails as ω≪Δ in (**a**); (**d**) Numerical simulation of the ground state population following multiple double crossings in a 16-qubit system.

To gain some intuition, consider a system initialized to the state |0〉 and driven through the avoided crossing twice (meaning, one period of s(t)). After the double crossing, the population of the state |1〉, denoted $P_{+}^{\left(2\right)}$, approaches 0 for both $\omega \ll \Delta^{2}/A$ and for ω≫A: if $\omega \ll \Delta^{2}/A$, the adiabatic condition holds; the system always remains in the instantaneous ground state and, thus, returns to |0〉. Furthermore, in the limit ω≫A, the propagator approaches unity and the state remains unperturbed. In intermediate cases, an interesting phenomenon occurs: in the first passage of the avoided crossing, the system transfers almost perfectly from the initial ground state to the final excited state. However, a tiny amplitude leaks to the orthogonal state. The populations of the excited state and the ground state gain different phases between the two crossings, and, finally, interfere again in the second crossing. $P_{+}^{\left(2\right)}$ is affected by this interference and oscillates with the periodicity of the control 2π/ω in what is known as Landau–Zener–Stückelberg oscillations [13,14,15] (See Figure 2).

In the ω≫Δ regime, one can use the rotating wave approximation (see [19], and Appendix B) to show that, with periodic drive, the system oscillates around the *x* axis in the Bloch sphere with frequency

(4) $$\Omega =\Delta \left|J_{0}\left(\frac{A}{\omega }\right)\right|.$$

The algorithm will fail when A/ω equals a root of the Bessel function $J_{0}$, where a coherent destruction of tunneling (CDT) occurs, and Ω=0 ([20], see also [17,19]). CDT was previously suggested as a method to control interactions in quantum systems [21,22,23] and we use these ideas in algorithm B.

### 2.2. Grover as a Two-Level System

Interestingly, the Grover Hamiltonian $H_{G}\left(t\right)$ with a periodic control function is closely related to $H_{\mathrm{LZS}}\left(t\right)$. The key to the mapping is the subspace $V=\mathrm{span}\left\{|u\rangle ,|y\rangle \right\}$, which is invariant to $H_{G}\left(s\right)$ for all *s*, as originally noted by Farhi and Gutmann [24] (see proof in Appendix C). Although *V* is isomorphic to the Hilbert space of a two-level system, one cannot map |u〉,|y〉 to |0〉,|1〉 trivially in $H_{\mathrm{LZS}}$, because the first pair is only approximately orthogonal. To overcome this problem, we define a new basis $|\bar{0}\rangle ,|\bar{1}\rangle$, exponentially close to |u〉 and |y〉, as stated in the following claim:

**Claim** **1.** *The projection of $H_{G}\left(s\left(t\right)\right)$ on V satisfies:*

(5) $$H_{G}\left(s\left(t\right)\right){|}_{V}=\frac{I_{2}}{2}+\left(s\left(t\right)-\frac{1}{2}\right)\sqrt{1-\Delta^{2}}{\bar{\sigma }}_{z}-\frac{\Delta }{2}{\bar{\sigma }}_{x},$$

*wherein Δ=〈y|u〉. The operators ${\bar{\sigma }}_{x},{\bar{\sigma }}_{z}$ act on the states*

(6) $$\begin{array}{cc} |\bar{0}\rangle & =\sqrt{\frac{1+\sqrt{1-\Delta^{2}}}{2}}|u\rangle -\sqrt{\frac{1-\sqrt{1-\Delta^{2}}}{2}}|u^{⊥}\rangle \\ |\bar{1}\rangle & =\sqrt{\frac{1-\sqrt{1-\Delta^{2}}}{2}}|u\rangle +\sqrt{\frac{1+\sqrt{1-\Delta^{2}}}{2}}|u^{⊥}\rangle , \end{array}$$

*wherein $|u^{⊥}\rangle :=\frac{|y\rangle -\Delta |u\rangle }{\sqrt{1-\Delta^{2}}}$ is the vector orthogonal to |u〉 in V.*

See Appendix D for the proof.

## 3. Results

### 3.1. Algorithm A

Algorithm A is an immediate corollary of Claim 1. The Hamiltonian $H_{G}$ with the control function s(t)=(1−Acos(ωt))/2 acts on *V* as an LZS Hamiltonian on the states $|\bar{0}\rangle ,|\bar{1}\rangle$. Since $|\bar{0}\rangle$ and $|\bar{1}\rangle$ are exponentially close to |u〉 and |y〉, respectively, evolving |u〉 by $H_{G}\left(s\left(t\right)\right)$ will cause the system to oscillate between the states close to |u〉 and |y〉 with frequency $\Omega =\Delta \left|J_{0}\left(\sqrt{1-\Delta^{2}}A/\omega \right)\right|$. Hence, such a driven Hamiltonian can solve the Grover problem in time $O(\sqrt{2^{n}})$, i.e., the same complexity as the optimal circuit and adiabatic models.

A careful analysis of LZS interferometry shows that the algorithm finds *y* for a wide range of A,ω. We require only ω≫Δ for the rotating wave approximation to hold. $J_{0}\left(\sqrt{1-\Delta^{2}}A/\omega \right)$ is a factor of the algorithm’s runtime, hence A/ω should not be large (for z≫1, $|J_{0}\left(z\right)|∼1/\sqrt{z}$), and not too close to the roots of $J_{0}$ as it will cause Ω to diminish by CDT. Note that none of these constraints requires prior knowledge of the gap Δ, other than an upper bound. Hence, the algorithm is robust to a multiplicative error of the Hamiltonian due to calibration errors, similar to previous approaches [25]. A different choice of parameters may improve the algorithm’s robustness to the variations in the total evolution time, as demonstrated in [26].

The limit A=0 yields maximal Ω, and corresponds to evolving by the time-independent Hamiltonian $H_{G}\left(s\right){|}_{s=1/2}=\frac{1}{2}\left(I_{2}-\Delta {\bar{\sigma }}_{x}\right)$, which we denote $H_{1\;/\;2}$. This is exactly the “analog” algorithm for the search problem by Farhi and Gutmann [24], and, more generally, a search by a quantum walk [25,27,28,29,30,31,32,33]. The Hamiltonian $H_{1\;/\;2}$ is the core of algorithms for the search problem: in the adiabatic algorithm [12], the Hamiltonian spends most of the time close to $H_{1\;/\;2}$, where the gap is minimal [25], while the original gate-model algorithm by Grover [5] can be seen as a simulation (or an approximation by Trotter formula [34]) of $H_{1\;/\;2}$ [24].

### 3.2. Algorithm B

**Figure 3:**
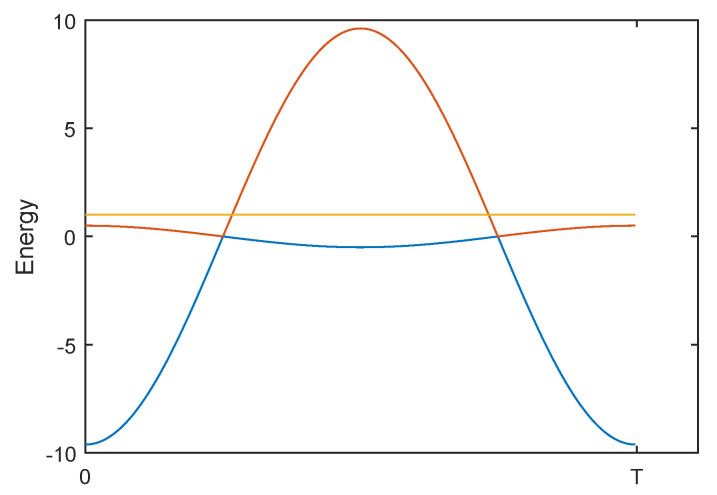
The spectrum of the noiseless $H_{B}$ over one period. The parameters are n=16,A=1,B=9.1193. Note that the yellow energy level is outside the invariant subspace *V*.

We now discuss adding an additional modulation to Algorithm A to improve its robustness without significantly increasing the runtime. Equation (7) defines the Hamiltonian for algorithm B, and the spectrum is illustrated in Figure 3.

(7) $$\begin{array}{cc} H_{B}\left(t\right) & =\left(I_{N}-|u\rangle \langle u|\right)\cdot \frac{1+A\cos(\omega t)}{2}+\left(I_{N}-|y\rangle \langle y|\right)\cdot \frac{1-A\cos(\omega t)}{2}-B\cos\left(\omega t\right)|u\rangle \langle u|\\ H_{B}{|}_{V} & =\left[\begin{array}{cc} \frac{1}{2}-\left(B+\frac{A}{2}\right)\cos\left(\omega t\right) & -\frac{\Delta }{2}\left(B\cos\left(\omega t\right)+1\right)\\ -\frac{\Delta }{2}\left(B\cos\left(\omega t\right)+1\right) & \frac{1}{2}+\frac{A}{2}\cos\left(\omega t\right) \end{array}\right]+O\left(\Delta^{2}\right). \end{array}$$

A natural question is whether Algorithm B is “cheating” by artificially increasing the gap or by manipulating resources. Here, a small detour discussing resources is in order. First, note that implementing the term $|u\rangle \langle u|$ requires no prior knowledge of *y*. Namely the algorithm is the same for all *y* (or *y* is “unknown”). This means that the total time wherein $H_{p}$ is active would have to be at least $2^{n/2}$. Otherwise, this would contradict the optimality of Grover’s algorithm [35]. To understand the role of *B*, one can partition $H_{B}$ by the Trotter approximation to slices of time independent Hamiltonians, where evolution by $H_{p}$ and by terms that are not $H_{p}$ alternate. In this picture, increasing $\left|B\right|$ corresponds to using a stronger quantum computer between calls to the black box, but has no effect on the query complexity of the problem (the total time $H_{p}$ is active).

### 3.3. Robustness Comparison

In what follows, we compare the robustness to control errors of algorithm B versus applying the time-independent Hamiltonian $H_{1\;/\;2}$, which also represents the standard gate model and adiabatic algorithms (for an analysis of the Hamiltonian-based search algorithm under different noise models, cf. [25,36,37]).

Hamiltonian control errors are uncontrolled terms causing the system to deviate unitarily from the intended evolution. The first error we focus on is in the form $A_{1}\cos\left(\omega_{1}t+\varphi \right){\bar{\sigma }}_{z}$ that preserves the subspace *V* and represents an error in s(t) (see Equation (5)).

Consider $H_{1\;/\;2}$ with a harmonic control error in s(t):

(8) $${\tilde{H}}_{1\;/\;2}=\frac{I_{2}}{2}-\frac{\Delta }{2}{\bar{\sigma }}_{x}+A_{1}\cos\left(\omega_{1}t+\varphi \right){\bar{\sigma }}_{z}.$$

This is exactly the LZS Hamiltonian. Therefore, for high frequency errors ($\omega_{1}\gg \Delta$), the Rabi frequency is $\tilde{\Omega }=\Delta \left|J_{0}\left(\frac{A_{1}}{\omega_{1}}\right)\right|$, and the evolution is generally unaffected. On the other hand, for $\omega_{1}=0$, even $A_{1}\approx \Delta$ may cause the system to freeze in the initial state because the ${\bar{\sigma }}_{z}$ rotation may become more dominant than the desired ${\bar{\sigma }}_{x}$ rotation. Hence, algorithms based on $H_{1\;/\;2}$ are not robust to low frequency control errors.

**Figure 4:**
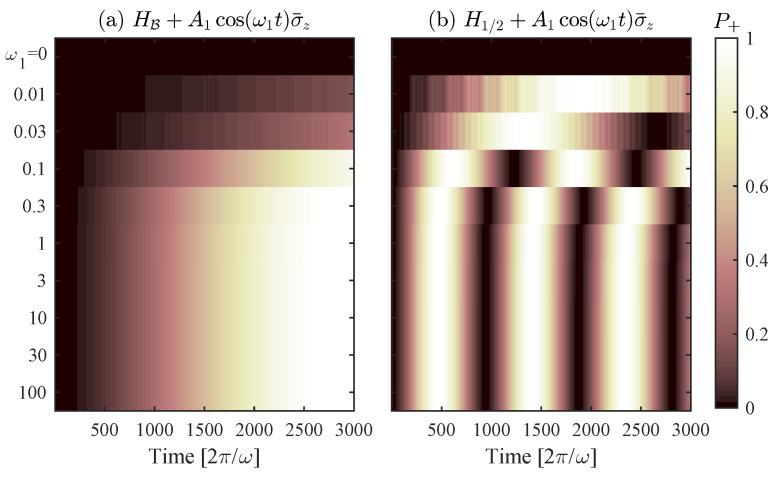
Sixteen-qubit numerical simulation comparing the robustness of Algorithm B versus an evolution by $H_{1\;/\;2}$: (**a**) Algorithm B with parameters ω=3.67,A=1,B=9.12; and (**b**) an evolution by $H_{1\;/\;2}$. The error $A_{1}\cos\left(\omega_{1}t\right){\bar{\sigma }}_{z}$ with $A_{1}=0.05$ is equivalent to an error in s(t). Each row in both panels is a simulation with different $\omega_{1}$ that is displayed on the y-axis. The brightness of the row changes from left to right as the value of $P_{+}$ varies in time under the noise of the specified $\omega_{1}$. Both algorithms are influenced by errors with $\omega_{1}\approx \Delta =0.125$, and fail as $\omega_{1}$ diminishes. However, both are generally robust to high frequency errors.

Algorithm B generally shows similar robustness (see Figure 4). It fails to find *y* when $\omega_{1}=0$ and $A_{1}\approx \Delta$ for the same reasons $H_{1\;/\;2}$ fails. For high frequency errors, we write the Hamiltonian $H_{B}+A_{1}\cos\left(\omega_{1}t+\varphi \right){\bar{\sigma }}_{z}$ in the appropriate rotating frame (around ${\bar{\sigma }}_{z}$) while neglecting $O(\Delta^{2})$ terms:

$$\begin{array}{cc} \; & {\tilde{H}}_{B}^{\prime }{|}_{V}=\left[\begin{array}{cc} 0 & -\frac{\Delta }{2}\left(B\cos\left(\omega t\right)+1\right)\chi \\ \;\\ -\frac{\Delta }{2}\left(B\cos\left(\omega t\right)+1\right)\chi^{*} & 0 \end{array}\right]\\ \; & \chi =\sum_{k,k_{1}=-\infty }^{\infty }J_{k}\left(\frac{A+B}{\omega }\right)J_{k_{1}}\left(\frac{2A_{1}}{\omega_{1}}\right)e^{ik_{1}\left(\omega_{1}t+\varphi \right)-ik\omega t}. \end{array}$$

See Appendix E for details.

The algorithm is generally unaffected by high frequency errors ($\omega_{1}\gg \Delta$) where all terms except $k=k_{1}=0$ average out, and the Rabi oscillation is $\tilde{\Omega }=J_{0}\left(\frac{A+B}{\omega }\right)J_{0}\left(\frac{2A_{1}}{\omega_{1}}\right)$. Note that, if $k_{1}\omega_{1}\approx k\omega$ for some values of $k,k_{1}$, then these terms would not average out, and may, in principle, cause the algorithm to fail because of CDT.

The second error we consider in our comparison are errors that do not preserve *V*. For their analysis, we use a three-level system toy model, composed of the previously defined states $|\bar{0}\rangle ,|\bar{1}\rangle$ and an additional state $|\bar{2}\rangle$, which represents a state outside of *V*. The error term we choose to focus on is the term $\eta (|\bar{0}\rangle \langle \bar{2}|+|\bar{2}\rangle \langle \bar{0}|)$. The Hamiltonians take the form:

(9) $$\begin{array}{c} H_{1\;/\;2}=\left[\begin{array}{ccc} \frac{1}{2} & -\frac{\Delta }{2} & \eta \\ -\frac{\Delta }{2} & \frac{1}{2} & 0\\ \eta & 0 & 1 \end{array}\right]\;\;H_{B}=\left[\begin{array}{ccc} \frac{1}{2}-\left(B+\frac{A}{2}\right)\cos\left(\omega t\right) & -\frac{\Delta }{2}\left(B\cos\left(\omega t\right)+1\right) & \eta \\ -\frac{\Delta }{2}\left(B\cos\left(\omega t\right)+1\right) & \frac{1}{2}+\frac{A}{2}\cos\left(\omega t\right) & 0\\ \eta & 0 & 1 \end{array}\right]. \end{array}$$

Interestingly, $H_{1\;/\;2}$ already has some inherent robustness to errors diverting the system to $|\bar{2}\rangle$: the diagonal elements of $H_{1\;/\;2}$ can be considered as “potential energies” of three sites. Therefore, a particle in $|\bar{0}\rangle$ needs to overcome a potential difference to reach $|\bar{2}\rangle$, while it does not need to face a barrier when transitioning to $|\bar{1}\rangle$.

**Figure 5:**
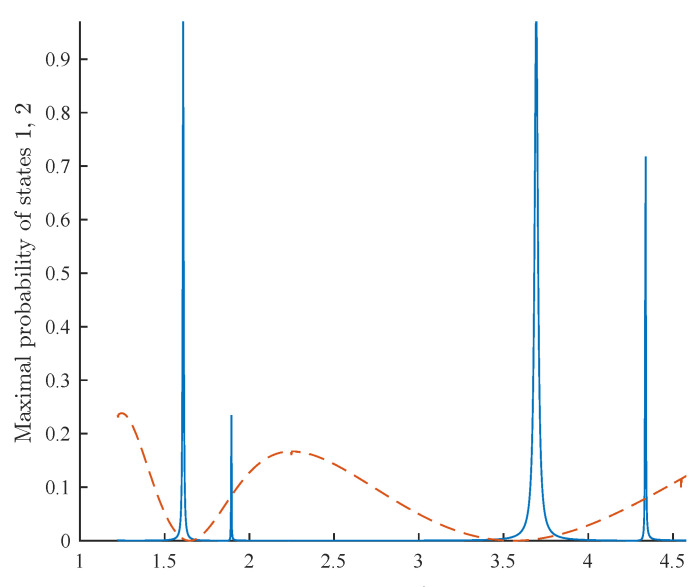
A simulation of Algorithm B with the control errors which do not preserve *V*, as expressed in Equation (9). We set n=20,A=1,B=9.12,η=0.3, and simulated the three-level system with different values of ω (*x* axis). For every simulation, two data points were plotted for the maximal probability reached by the states $\bar{1}$ (blue, solid) and $\bar{2}$ (orange, dashed) in the time interval t=[0,150/Δ]. The ratio between the desired transition $\Delta /2\approx 5\times 10^{-4}$ and the control error η is 1:600; evolving $|\bar{0}\rangle$ by $H_{1\;/\;2}$ yields $|\bar{1}\rangle$ with probability $3.2\times 10^{-5}$. The graph shows that for some ω, the peak probability of $\bar{1}$ is close to one. Hence, Algorithm B is more robust to such errors. Note that Equation (10) predicts that the transition $\bar{0}\to \bar{1}$ peaks for ω≈1.74,4, which corresponds for the first two roots of $J_{0}\left(\frac{B+A/2}{\omega }\right)$, where the $\bar{0}\to \bar{2}$ transition is strongly suppressed. The simulation shows that the desired transition $\bar{0}\to \bar{1}$ peaks at two frequencies around each root—this implies that the rotating wave approximation is insufficient to describe the dynamics of the system.

Algorithm B improves the natural error suppression by adding CDT between the states $|\bar{0}\rangle$ and $|\bar{2}\rangle$, while allowing transitions between $|\bar{0}\rangle$ and $|\bar{1}\rangle$. We hereby present a simplified analysis, using the rotating wave approximation. However, we stress that finer tools, such as Floquet theory [38], better describe the dynamics of the system and should be used when one attempts to find optimal values for B,A,ω (see Figure 5). After changing to a rotating frame where $\langle \bar{0}|H_{B}|\bar{0}\rangle =\langle \bar{1}|H_{B}|\bar{1}\rangle =0$, and using the rotating wave approximation, we have:

(10) $$H_{B}^{\prime }=\left[\begin{array}{ccc} 0 & -\frac{\Delta }{2}J_{0}\left(\frac{B+A}{\omega }\right) & \eta J_{0}\left(\frac{B+A/2}{\omega }\right)\\ -\frac{\Delta }{2}J_{0}\left(\frac{B+A}{\omega }\right) & 0 & 0\\ \eta J_{0}\left(\frac{B+A/2}{\omega }\right) & 0 & \frac{1}{2} \end{array}\right].$$

Note that, in Equation (10), the argument for the Bessel function in $\langle \bar{0}|H_{B}|\bar{1}\rangle$ is different from the one in $\langle \bar{0}|H_{B}|\bar{2}\rangle$. By choosing $\frac{B+A/2}{\omega }$ to be a root of $J_{0}$, the transition from $|\bar{0}\rangle$ to $|\bar{2}\rangle$ is suppressed. On the other hand, the term $\left|\langle \bar{0}|H_{B}|\bar{1}\rangle \right|$, which dominates Ω and the computation time, is multiplied by a factor of $J_{0}\left(\frac{B+A}{\omega }\right)\neq 0$. Namely, the runtime is increased by a factor of $1/J_{0}\left(\frac{B+A}{\omega }\right)$, while the error term η is suppressed. Our numerical simulations (see Figure 5) confirm that, for a given scenario, the probability of an evolution $H_{1\;/\;2}$ to find *y* is $3.2\times 10^{-5}$, while, with tuned parameters, Algorithm B finds *y* with near certainty.

*Thermal noise:* Implementing error correction for quantum algorithms based on continuous Hamiltonians instead of discrete gates is an open problem [39]. One can suppress thermal noise (as well as control errors) by encoding the Hamiltonian by a stabilizer code [40], combined with dynamical decoupling [41], energy gap protection [42], or Zeno effect suppression [43]. All of them function very similarly [39,44], providing enhanced performance for finite size systems, which were recently described as noisy intermediate scale quantum (NISQ) [45]. For an exponential time algorithm, such as the unstructured search problem, error suppression methods ultimately fail, and an active error correction is required.

## 4. Discussion

In this work, we propose a new diabatic algorithm for solving the Grover problem using LZS interferometry. Diabatic scheduling for computation was researched recently [46,47] and, in some cases, even showed superior performances to adiabatic scheduling. Variational Quantum Algorithms (VQA) [48,49,50,51] and Quantum Approximate Optimization Algorithms (QAOA) [52,53], in which the path is optimized by a classical computer, can also be considered diabatic. However, our work shows a new application of diabaticity: suppression of control errors. We conjecture the need for hybrid algorithms (diabatic/adiabatic), tailored to the noise parameters of a system.

One possible near term implementation of such model is the unstructured search problem on the hypercube [30,54], which exhibits an avoided crossing between the two lowest eigenvalues, while the rest of the spectrum is separated. Driving the system with oscillations that are slow with respect to the gap between the first and second excited states will cause the two lowest energy states to act as a two-level system.

While the Grover problem is important on its own, it is interesting to examine the applicability of our paradigm to additional problems. It remains an open question whether one can translate any adiabatic algorithm to a diabatic algorithm.

Finally, it is interesting to find an expression for the optimal driving frequencies in Algorithm B, including their spectral width, and effectiveness (based on a full numerical analysis of the Floquet problem).

## Appendix A. Dimensionless Units

In the main text we have worked in dimensionless units, specially because computational/query complexity is invariant to multiplicative factors. Here, we rewrite the main results while keeping the units consistent. The problem Hamiltonian is normally given with an energy scale ε (ℏ=1):

(A1) $$H_{p}=\varepsilon \left(I_{N}-|y\rangle \langle y|\right)$$

The Hamiltonian corresponding to Algorithm A is the following:

(A2) $$\begin{array}{cc} H_{G}\left(s(t)\right) & =\varepsilon \left[\left(1-s\left(t\right)\right)\cdot \left(I_{N}-|u\rangle \langle u|\right)+s\left(t\right)\cdot \left(I_{N}-|y\rangle \langle y|\right)\right]\\ s(t) & =\frac{1-a\cos(\omega t)}{2}, \end{array}$$

where a∈[0,1] is the dimensionless amplitude of the control function s(t). Note that the minimal energy gap is $\Delta =2^{-n/2}\varepsilon$. Claim A1 takes the following form:
**Claim** **A1.** *The projection of $H_{G}\left(s\left(t\right)\right)$ on V satisfies:*

(A3) $$\begin{array}{cc} H_{G}\left(s\left(t\right)\right){|}_{V} & =\varepsilon \left[\frac{I_{2}}{2}+\left(s\left(t\right)-\frac{1}{2}\right)\sqrt{1-\delta^{2}}{\bar{\sigma }}_{z}-\frac{\delta }{2}{\bar{\sigma }}_{x}\right]\\ \; & =\varepsilon \left[\frac{I_{2}}{2}+\left(\frac{-a\cos(\omega t)}{2}\right)\sqrt{1-\delta^{2}}{\bar{\sigma }}_{z}-\frac{\delta }{2}{\bar{\sigma }}_{x}\right] \end{array}$$

*wherein δ=〈y|u〉 is dimensionless. The operators ${\bar{\sigma }}_{x},{\bar{\sigma }}_{z}$ act on the states*

(A4) $$\begin{array}{cc} |\bar{0}\rangle & =\sqrt{\frac{1+\sqrt{1-\delta^{2}}}{2}}|u\rangle -\sqrt{\frac{1-\sqrt{1-\delta^{2}}}{2}}|u^{⊥}\rangle \\ |\bar{1}\rangle & =\sqrt{\frac{1-\sqrt{1-\delta^{2}}}{2}}|u\rangle +\sqrt{\frac{1+\sqrt{1-\delta^{2}}}{2}}|u^{⊥}\rangle \end{array}$$

*wherein $|u^{⊥}\rangle :=\frac{|y\rangle -\delta |u\rangle }{\sqrt{1-\delta^{2}}}$ is the vector orthogonal to |u〉 in V.*

The rotating frame approximation holds when $\omega \gg \Delta =2^{-n/2}\varepsilon$. The run time of the algorithm in this case is inversely proportional to the Rabi frequency $\Omega =\varepsilon \delta \cdot J_{0}\left(\frac{\sqrt{1-\delta^{2}}a\varepsilon }{\omega }\right)$. On the other hand, when $\varepsilon a\omega \ll \Delta^{2}$, the process is adiabatic.

Algorithm B is defined using an additional dimensionless variable *b*:

(A5) $$\begin{array}{cc} H_{B}\left(t\right) & =\varepsilon \left(\left(I_{N}-|u\rangle \langle u|\right)\cdot \frac{1+a\cos(\omega t)}{2}+\left(I_{N}-|y\rangle \langle y|\right)\cdot \frac{1-a\cos(\omega t)}{2}-b\cos\left(\omega t\right)|u\rangle \langle u|\right)\\ H_{B}{|}_{V} & =\varepsilon \left[\begin{array}{cc} \frac{1}{2}-\left(b+\frac{a}{2}\right)\cos\left(\omega t\right) & -\frac{\delta }{2}\left(b\cos\left(\omega t\right)+1\right)\\ -\frac{\delta }{2}\left(b\cos\left(\omega t\right)+1\right) & \frac{1}{2}+\frac{a}{2}\cos\left(\omega t\right) \end{array}\right]+\varepsilon \left(\left|a\right|+\left|b\right|\right)\cdot O\left(\delta^{2}\right), \end{array}$$

and by adding a unitary error from *V* to $V^{⊥}$ it takes the form:

(A6) $$\begin{array}{cc} \; & H_{B}=\varepsilon \left[\begin{array}{ccc} \frac{1}{2}-\left(b+\frac{a}{2}\right)\cos\left(\omega t\right) & -\frac{\delta }{2}\left(b\cos\left(\omega t\right)+1\right) & \eta \\ -\frac{\delta }{2}\left(b\cos\left(\omega t\right)+1\right) & \frac{1}{2}+\frac{a}{2}\cos\left(\omega t\right) & 0\\ \eta & 0 & 1 \end{array}\right]+\varepsilon \left(\left|a\right|+\left|b\right|\right)\cdot O\left(\delta^{2}\right). \end{array}$$

Finally, the optimal values for a,b,ω are in proximity to the roots of $J_{0}\left(\frac{\varepsilon (b+a/2)}{\omega }\right)$.

## Appendix B. Analysis of LZS Oscillations Using the Rotating Wave Approximation

In this section, we analyze the LZS oscillations and the robustness to errors by generalizing the rotating wave approximation analysis by [19,55].

**Claim** **A2.**
*The Rabi frequency of a system driven by $H_{\mathrm{LZS}}$ is*

(A7) $$\Omega =\Delta \left|J_{0}\left(\frac{A}{\omega }\right)\right|$$

**Proof.** We start with a two-level system and a general control function:

(A8) $$H\left(t\right):=\frac{1}{2}\left(-a\left(t\right)\sigma_{z}-\Delta \sigma_{x}\right)=\frac{1}{2}\left[\begin{array}{cc} -a(t) & -\Delta \\ -\Delta & a(t) \end{array}\right].$$

Changing to the rotating frame yields

(A9) $$|\psi (t)\rangle =U(t)|\psi^{\prime }\left(t\right)\rangle ,$$

wherein

(A10) $$U\left(t\right)=\exp\left\{\frac{i}{2}\sigma_{z}\int a\left(t\right)dt\right\}.$$

Note that the populations of the ground state and the excited state are invariant to this transformation. The effective Hamiltonian $H^{\prime }$, which satisfies the Shrödinger equation in the rotating frame, $i\frac{d}{dt}|\psi^{\prime }\rangle =H^{\prime }|\psi^{\prime }\rangle ,$ is the following [56]:

(A11) $$H^{\prime }\left(t\right)=U^{†}\left(t\right)H\left(t\right)U\left(t\right)-iU^{†}\left(t\right)\frac{dU(t)}{dt}=-\frac{\Delta }{2}\left[\begin{array}{cc} 0 & e^{-i\int a(t)dt}\\ e^{i\int a(t)dt} & 0 \end{array}\right].$$

By assigning a(t)=Acos(ωt), integrating and using the Bessel–Anger identity

(A12) $$\exp\left\{iz\sin\gamma \right\}=\sum_{k=-\infty }^{\infty }J_{k}\left(z\right)e^{ik\gamma },$$

we get

(A13) $$\begin{array}{cc} H^{\prime }\left(t\right) & =-\frac{\Delta }{2}\left[\begin{array}{cc} 0 & \exp\left\{-i\frac{A}{\omega }\sin\left(\omega t\right)\right\}\\ \exp\left\{i\frac{A}{\omega }\sin\left(\omega t\right)\right\} & 0 \end{array}\right]\\ \; & =-\frac{\Delta }{2}\left[\begin{array}{cc} 0 & \sum_{k=-\infty }^{\infty }J_{k}\left(A/\omega \right)e^{-ik\omega t}\\ \sum_{k=-\infty }^{\infty }J_{k}\left(A/\omega \right)e^{ik\omega t} & 0 \end{array}\right]. \end{array}$$

A general pulse shape was analyzed in [57]. Since ω≫Δ, we can use the rotating wave approximation, which yields

(A14) $$H^{\prime }\left(t\right)=-\frac{\Delta }{2}\left[\begin{array}{cc} 0 & J_{0}\left(A/\omega \right)\\ J_{0}\left(A/\omega \right) & 0 \end{array}\right],$$

and the proof follows. □

## Appendix C. Invariant Subspace in *H_G_*(*s*)

**Claim** **A3** (Following Farhi and Gutmann [24])**.** *The subspace V=span{|y〉,|u〉} is invariant to*

(A15) $$H_{G}\left(s(t)\right)=\left(1-s\left(t\right)\right)\cdot \left(I_{N}-|u\rangle \langle u|\right)+s\left(t\right)\cdot \left(I_{N}-|y\rangle \langle y|\right).$$

**Proof.** A general vector in *V* takes the form $|v\rangle =\left(\alpha |u\rangle +\beta |y\rangle \right)\in V$. When $H_{G}\left(s\right)$ acts on |u〉 and |y〉, we have

(A16) $$H_{G}\left(s\left(t\right)\right)|u\rangle =s\left(t\right)\left(|u\rangle -\langle u|y\rangle |y\rangle \right)\in V$$

(A17) $$H_{G}\left(s\left(t\right)\right)|y\rangle =\left(1-s\left(t\right)\right)\left(|y\rangle -\langle y|u\rangle |u\rangle \right)\in V.$$

Therefore, $H_{G}\left(s\left(t\right)\right)|v\rangle \in V$ for any choice of s,α,β. □

This invariance allows us to reduce the $2^{n}$-dimensional problem to a 2-dimensional problem as required for the similarity relation in Claim 1. Additionally, this enables numerical simulations for high values of *n*.

## Appendix D. Proof of Claim 1

Here, we show the similarity of $H_{G}$ in the subspace *V* to the LZS Hamiltonian. It is clear that |u〉,|y〉 are not orthogonal and therefore they cannot be trivially mapped to |0〉,|1〉 in $H_{\mathrm{LZS}}$. To overcome the problem, we find a basis that is exponentially close to |u〉,|y〉, which allows stating the similarity relation (clearly these corrections are relevant only for small and intermediate size systems). Note that the rate s(t) is also slightly adjusted.

**Claim** **A4.**
*The projection of $H_{G}\left(s\left(t\right)\right)$ on V satisfies:*

(A18) $$H_{G}\left(s\left(t\right)\right){|}_{V}=\left(\frac{I_{2}}{2}+\left(s\left(t\right)-\frac{1}{2}\right)\sqrt{1-\Delta^{2}}{\bar{\sigma }}_{z}-\frac{\Delta }{2}{\bar{\sigma }}_{x}\right)$$

*wherein Δ=〈y|u〉. The operators ${\bar{\sigma }}_{x},{\bar{\sigma }}_{z}$ act on the states*

(A19) $$\begin{array}{cc} |\bar{0}\rangle & =\sqrt{\frac{1+\sqrt{1-\Delta^{2}}}{2}}|u\rangle -\sqrt{\frac{1-\sqrt{1-\Delta^{2}}}{2}}|u^{⊥}\rangle \\ |\bar{1}\rangle & =\sqrt{\frac{1-\sqrt{1-\Delta^{2}}}{2}}|u\rangle +\sqrt{\frac{1+\sqrt{1-\Delta^{2}}}{2}}|u^{⊥}\rangle \end{array}$$

*wherein $|u^{⊥}\rangle :=\frac{|y\rangle -\Delta |u\rangle }{\sqrt{1-\Delta^{2}}}$ is the vector orthogonal to |u〉 in V.*

**Proof.** By definition,

(A20) $$H_{G}\left(s(t)\right)=\left(1-s\left(t\right)\right)\cdot \left(I_{N}-|u\rangle \langle u|\right)+s\left(t\right)\cdot \left(I_{N}-|y\rangle \langle y|\right).$$

We wish to prove that the matrix form of $H_{G}\left(s\left(t\right)\right)$ projected on *V*, in the basis $|\bar{0}\rangle ,|\bar{1}\rangle$ is:

(A21) $$H_{G}\left(s\left(t\right)\right){|}_{V}=\left(\begin{array}{cc} \frac{1}{2}+\left(s\left(t\right)-\frac{1}{2}\right)\xi & -\frac{\Delta }{2}\\ -\frac{\Delta }{2} & \frac{1}{2}-\left(s\left(t\right)-\frac{1}{2}\right)\xi . \end{array}\right)$$

wherein $\xi =\sqrt{1-\Delta^{2}}$. In other words, we should prove that

(A22) $$\begin{array}{cc} \langle \bar{0}|H_{G}\left(s\left(t\right)\right)|\bar{0}\rangle & =\frac{1}{2}+\left(s\left(t\right)-\frac{1}{2}\right)\xi \\ \langle \bar{1}|H_{G}\left(s\left(t\right)\right)|\bar{1}\rangle & =\frac{1}{2}-\left(s\left(t\right)-\frac{1}{2}\right)\xi \\ \langle \bar{0}|H_{G}\left(s\left(t\right)\right)|\bar{1}\rangle & =-\frac{\Delta }{2} \end{array}$$

It is helpful to use the following three equations:

(A23) $$\begin{array}{cc} \langle u|H_{G}\left(s\left(t\right)\right)|u\rangle & =\left(1-\Delta^{2}\right)s\left(t\right)\\ \langle u^{⊥}|H_{G}\left(s\left(t\right)\right)|u\rangle & =-\Delta \xi \cdot s(t)\\ \langle u^{⊥}|H_{G}\left(s\left(t\right)\right)|u^{⊥}\rangle & =1-\xi^{2}\cdot s\left(t\right). \end{array}$$

Calculating the matrix elements:

(A24) $$\begin{array}{cc} \langle \bar{0}|H_{G}\left(s\left(t\right)\right)|\bar{0}\rangle & =\frac{1+\xi }{2}\cdot \left(1-\Delta^{2}\right)s\left(t\right)+2\Delta \xi s\left(t\right)\sqrt{\frac{1+\xi }{2}\cdot \frac{1-\xi }{2}}+\left(1-\xi^{2}s\left(t\right)\right)\frac{1-\xi }{2}\\ \; & =\xi^{2}\frac{1+\xi }{2}s\left(t\right)+\Delta^{2}\xi s\left(t\right)+\left(1-\xi^{2}s\left(t\right)\right)\frac{1-\xi }{2}\\ \; & =s\left(t\right)\cdot \left(\xi^{2}\frac{1+\xi }{2}+\left(1-\xi^{2}\right)\xi -\xi^{2}\frac{1-\xi }{2}\right)+\frac{1-\xi }{2}=\frac{1}{2}+\left(s\left(t\right)-\frac{1}{2}\right)\xi \end{array}$$

(A25) $$\begin{array}{cc} \langle \bar{1}|H_{G}\left(s\left(t\right)\right)|\bar{1}\rangle \; & =\frac{1-\xi }{2}\cdot \left(1-\Delta^{2}\right)s\left(t\right)-2\Delta \xi s\left(t\right)\sqrt{\frac{1+\xi }{2}\cdot \frac{1-\xi }{2}}+\left(1-\xi^{2}s\left(t\right)\right)\frac{1+\xi }{2}\\ \; & =\xi^{2}\frac{1-\xi }{2}s\left(t\right)-\Delta^{2}\xi s\left(t\right)+\left(1-\xi^{2}s\left(t\right)\right)\frac{1+\xi }{2}\\ \; & =s\left(t\right)\left(\xi^{2}\frac{1-\xi }{2}-\left(1-\xi^{2}\right)\xi -\xi^{2}\frac{1+\xi }{2}\right)+\frac{1+\xi }{2}=\frac{1}{2}-\left(s\left(t\right)-\frac{1}{2}\right)\xi \end{array}$$

(A26) $$\begin{array}{cc} \langle \bar{1}|H_{G}\left(s\left(t\right)\right)|\bar{0}\rangle \; & =\left(1-\Delta^{2}\right)s\left(t\right)\sqrt{\frac{\left(1+\xi )(1-\xi \right)}{4}}+\Delta \xi \cdot s\left(t\right)\left(\frac{1-\xi }{2}-\frac{1+\xi }{2}\right)\\ \; & -\left(1-\xi^{2}\cdot s\left(t\right)\right)\sqrt{\frac{\left(1-\xi )(1+\xi \right)}{4}}\\ \; & =\frac{\xi^{2}\Delta }{2}s\left(t\right)-\Delta \xi^{2}s\left(t\right)-\left(1-\xi^{2}\cdot s\left(t\right)\right)\frac{\Delta }{2}=-\frac{\Delta }{2} \end{array}$$

The proof of Claim 1 follows. □

## Appendix E. Analysis of Algorithm B Using the Rotating Wave Approximation

We add detailed derivation of $H_{B}$ (following Ashhab et al. [19]). In the case of ${\bar{\sigma }}_{z}$ error,
**Claim** **A5.** *Let*

(A27) $${\tilde{H}}_{B}{|}_{V}=\left[\begin{array}{cc} \frac{1}{2}-\left(B+\frac{A}{2}\right)\cos\left(\omega t\right) & -\frac{\Delta }{2}\left(B\cos\left(\omega t\right)+1\right)\\ -\frac{\Delta }{2}\left(B\cos\left(\omega t\right)+1\right) & \frac{1}{2}+\frac{A}{2}\cos\left(\omega t\right) \end{array}\right]+A_{1}{\bar{\sigma }}_{z}\cos\left(\omega_{1}t+\varphi \right)+O\left(\Delta^{2}\right).$$

*Using a rotation around ${\bar{\sigma }}_{z}$ the effective Hamiltonian takes the form:*

(A28) $$\begin{array}{cc} {\tilde{H}}_{B}^{\prime }{|}_{V} & =\left[\begin{array}{cc} 0 & -\frac{\Delta }{2}\left(B\cos\left(\omega t\right)+1\right)\chi \\ \;\\ -\frac{\Delta }{2}\left(B\cos\left(\omega t\right)+1\right)\chi^{*} & 0 \end{array}\right]+O\left(\Delta^{2}\right)\\ \chi & =\sum_{k,k_{1}=-\infty }^{\infty }J_{k}\left(\frac{A+B}{\omega }\right)J_{k_{1}}\left(\frac{2A_{1}}{\omega_{1}}\right)e^{ik_{1}\left(\omega_{1}t+\varphi \right)-ik\omega t} \end{array}$$

**Proof.** We subtract the global (time dependent) energy offset $\frac{1}{2}-B/2\cos\left(\omega t\right)$. Terms of order $O(\Delta^{2})$ can be neglected because the Hamiltonian is applied for duration O(1/Δ). We get

(A29) $${\tilde{H}}_{B}{|}_{V}=\left(-\frac{B+A}{2}\cos\left(\omega t\right)+A_{1}\cos\left(\omega_{1}t+\varphi \right)\right){\bar{\sigma }}_{z}-\frac{\Delta }{2}\left(B\cos\left(\omega t\right)+1\right){\bar{\sigma }}_{x}$$

Next, we choose a rotating frame, in which the diagonal is zero, in a similar way to Equation (A11), but with $a\left(t\right)=\left(A+B\right)\cos\left(\omega t\right)-2A_{1}\cos\left(\omega_{1}t+\varphi \right)$:

(A30) $$\begin{array}{cc} {\tilde{H}}_{B}^{\prime }{|}_{V} & =\left[\begin{array}{cc} 0 & -\frac{\Delta }{2}\left(B\cos\left(\omega t\right)+1\right)e^{-i\int a(t)dt}\\ -\frac{\Delta }{2}\left(B\cos\left(\omega t\right)+1\right)e^{i\int a(t)dt} & 0 \end{array}\right]\\ \; & =-\frac{{\bar{\sigma }}_{+}}{2}\cdot \frac{\Delta }{2}\left(B\cos\left(\omega t\right)+1\right)\exp\left\{-i\left(\frac{A+B}{\omega }\sin\left(\omega t\right)-\frac{2A_{1}}{\omega_{1}}\sin\left(\omega_{1}t+\varphi \right)\right)\right\}+h.c.\\ \; & =-\frac{{\bar{\sigma }}_{+}}{2}\cdot \frac{\Delta }{2}\left(B\cos\left(\omega t\right)+1\right)\sum_{k,k_{1}=-\infty }^{\infty }J_{k}\left(\frac{A+B}{\omega }\right)e^{-ik\omega t}\cdot J_{k_{1}}\left(\frac{2A_{1}}{\omega_{1}}\right)e^{ik_{1}\left(\omega_{1}t+\varphi \right)}+h.c., \end{array}$$

wherein ${\bar{\sigma }}_{+}={\bar{\sigma }}_{x}+i{\bar{\sigma }}_{y}$. The proof follows. □

Similarly, we derive the transformation of the three-level system in Equation (10).

**Claim** **A6.**
*Let*

(A31) $$H_{B}=\left[\begin{array}{ccc} \frac{1}{2}-\left(B+\frac{A}{2}\right)\cos\left(\omega t\right) & -\frac{\Delta }{2}\left(B\cos\left(\omega t\right)+1\right) & \eta \\ -\frac{\Delta }{2}\left(B\cos\left(\omega t\right)+1\right) & \frac{1}{2}+\frac{A}{2}\cos\left(\omega t\right) & 0\\ \eta & 0 & 1 \end{array}\right]$$

*By a changing to a frame, which preserves $|\bar{0}\rangle ,|\bar{1}\rangle ,|\bar{2}\rangle$, the Hamiltonian can be approximated by Equation (10):*

(A32) $$H_{B}^{\prime }=\left[\begin{array}{ccc} 0 & -\frac{\Delta }{2}J_{0}\left(\frac{B+A}{\omega }\right) & \eta J_{0}\left(\frac{B+A/2}{\omega }\right)\\ -\frac{\Delta }{2}J_{0}\left(\frac{B+A}{\omega }\right) & 0 & 0\\ \eta J_{0}\left(\frac{B+A/2}{\omega }\right) & 0 & \frac{1}{2} \end{array}\right].$$

**Proof.** First, we change the reference frame by the first equality of Equation (A11), with

(A33) $$U=\exp\left\{i\left(B+\frac{A}{2}\right)\frac{\sin(\omega t)}{\omega }|\bar{0}\rangle \langle \bar{0}|-i\frac{A\sin(\omega t)}{2\omega }|\bar{1}\rangle \langle \bar{1}|\right\}.$$

We get

(A34) $$H_{B}^{\prime }=\left[\begin{array}{ccc} \frac{1}{2} & -\frac{\Delta }{2}\left(B\cos\left(\omega t\right)+1\right)e^{-i\frac{A+B}{\omega }\sin\left(\omega t\right)} & \eta e^{-i\frac{B+A/2}{\omega }\sin\left(\omega t\right)}\\ -\frac{\Delta }{2}\left(B\cos\left(\omega t\right)+1\right)e^{i\frac{A+B}{\omega }\sin\left(\omega t\right)} & \frac{1}{2} & 0\\ \eta e^{i\frac{B+A/2}{\omega }\sin\left(\omega t\right)} & 0 & 1 \end{array}\right].$$

The diagonal can be adjusted by subtracting $\frac{1}{2}I$. By using the Bessel–Anger identity in Equation (A12), and by neglecting all but the zero frequency terms (rotating wave approximation), we get

(A35) $$H_{B}^{\prime }=\left[\begin{array}{ccc} 0 & -\frac{\Delta }{2}\left[J_{0}\left(\frac{B+A}{\omega }\right)+\frac{B\left[J_{1}\left(\frac{B+A}{\omega }\right)+J_{-1}\left(\frac{B+A}{\omega }\right)\right]}{2}\right] & \eta J_{0}\left(\frac{B+A/2}{\omega }\right)\\ -\frac{\Delta }{2}\left[J_{0}\left(\frac{B+A}{\omega }\right)+\frac{B\left[J_{1}\left(\frac{B+A}{\omega }\right)+J_{-1}\left(\frac{B+A}{\omega }\right)\right]}{2}\right] & 0 & 0\\ \eta J_{0}\left(\frac{B+A/2}{\omega }\right) & 0 & \frac{1}{2} \end{array}\right].$$

Finally, $J_{-1}\left(z\right)=-J_{1}\left(z\right)$ for all *z* and the proof follows. □
